# Correspondence: Compound 17b and formyl peptide receptor biased agonism in relation to cardioprotective effects in ischaemia-reperfusion injury

**DOI:** 10.1038/s41467-017-02654-2

**Published:** 2018-02-07

**Authors:** Agostino Cilibrizzi

**Affiliations:** Institute of Pharmaceutical Science, King’s College London, Stamford Street, London, SE1 9NH UK

In a recent publication^1^ Qin and co-workers report the biological evaluation of compound 17b^2^, also referred as (±)−5a^3^, a chiral small-molecule agonist of formyl peptide receptors (FPRs). By testing ligand potency in Chinese hamster ovary (CHO)-transfected cells, the authors determine that compound 17b (used as a racemic mixture) is a dual agonist for FPR1 and FPR2 isoforms. Moreover, a marked biased effect is observed with the racemate, with respect to the Ca^2+^ mobilisation response in cellulo. Based on these findings, the authors conclude that the FPR bias caused by compound 17b reflects a number of cardioprotective benefits in ischaemia–reperfusion injury (I–R) in vivo. The present comment analyses why these results differ from previous reports in terms of FPR potency/apparent selectivity and why the relevance of chirality must be assessed in order to establish if FPR bias is occurring.

Human FPRs are G-protein-coupled receptors (GPCRs) that mediate host defence in a variety of inflammatory-based pathological conditions^4,5^. Compound 17b^2^ is a chiral member of the 2-arylacetamido-pyridazinone class of FPR agonists^2–4,6^, which was initially reported to demonstrate FPR1 selectivity as a racemate^2^. As is often the case with GPCR testing, cell-type discrepancies are observed and the biological profile of such a ligand (indirectly established through its potency) can vary, depending on the cell line investigated^7,8^. Potency values (i.e. EC_50_) are typically evaluated through the indirect measurement of second messenger levels. This is the most common approach in GPCR research^8,9^. Quite different response patterns to agonists and/or antagonists can be found, for instance each signalling pathway can contribute its own biological effect depending on the unique receptor–ligand complex formed in the particular cell. In the same way, various FPR ligands have been shown not to bind to a particular receptor subtype with the same potency when a functional assay is performed in different transfected cell lines (e.g. CHO, RBL-2H3, HL-60) and under different experimental settings^10,11^. An example of this lack of uniformity in ligand recognition data is reported by Qin and collaborators^1^, where compound 17b was established as a FPR1/FPR2 dual agonist in FPR-transfected CHO cells, which is a different cellular context to that adopted in previous investigations^2,3^. In line with this report, Table 1 shows the results obtained with 15 known ligands^2,6^ of the same class (including compound 17b) in two different cell lines (HL-60 and RBL-2H3). Clearly, “low micromolar agonists” in one cell line may behave as “high micromolar agonists” in the other cell line (e.g. 14d, 14m, 14n, 14p). Furthermore, selective compounds for either FPR1 or FPR2 in one cell line behave as dual ligands in the other cell line (e.g. 14e, 14j, 6e, 11l, 17a, 33). Compounds 14f and 6d (Table 1) show the most impressive differences, being specific FPR1 agonists in HL-60 cells and completely devoid of activity in RBL-2H3 cells.

**Table 1:** Biological activity evaluation (i.e. agonism, through Ca^2+^ mobilisation) of selected ligands for FPR1 and FPR2 in HL-60 and RBL-2H3 transfected cells

n^a^	Ca^2+^ mobilisation—EC_50_, μM; efficacy (%)^b^				Conclusion on the basis of EC_50_ results in HL-60 cells	Conclusion on the basis of EC_50_ results in RBL-2H3 cells
	HL-60 cells^c^		RBL-2H3 cells^d^			
	FPR1	FPR2	FPR1	FPR2		
**14d** ^2^	2.6 (110)	4.0 (35)	44.7	21.1	Active for FPR1 and FPR2	LA for FPR1 and FPR2
**14e** ^2^	2.8 (90)	6.8 (40)	NA	1.8 (70)	Active for FPR1 and FPR2	Selective/active for FPR2
**14f** ^2^	7.6 (40)	NA	NA	NA	Selective for FPR1	NA
**14j** ^2^	7.7 (65)	14.4 (35)	1.8 (70)	NA	Active for FPR1/MA for FPR2	Selective for FPR1
**14l** ^2^	15.5 (25)	16.8 (25)	NA	51.4	MA for FPR1 and FPR2	Selective (LA) for FPR2
**14m** ^2^	2.3 (50)	NA	33.2	NA	Selective for FPR1	Selective (LA) for FPR1
**14n** ^2^	5.7 (50)	8.8 (95)	36.5	51.0	Active for FPR1 and FPR2	LA for FPR1 and FPR2
**14p** ^2^	10.5 (60)	12.3 (55)	35.4	38.3	MA for FPR1 and FPR2	LA for FPR1 and FPR2
**6d** ^6^	10.8 (80)	NA	NA	NA	Selective (MA) for FPR1	NA
**6e** ^6^	9.0 (110)	4.3 (25)	3.3 (105)	NA	Active for FPR1 and FPR2	Selective for FPR1
**11j** ^6^	4.5 (100)	14.1 (65)	1.4 (70)	2.8 (90)	Active for FPR1/MA for FPR2	Active for FPR1 and FPR2
**11l** ^6^	13.8 (20)	NA	3.6 (25)	3.8 (50)	Selective (MA) for FPR1	Active for FPR1 and FPR2
**17a** ^2^	9.7 (30)	5.4 (25)	NA	15.1 (75)	Active for FPR1 and FPR2	Selective (MA) for FPR2
**17b** ^2^	3.2 (90)	1.9 (20)^e^	1.6 (100)	3.2 (60)	Active for FPR1 and FPR2	Active for FPR1 and FPR2
**33** ^6^	11.2 (55)	NA	6.3 (100)	2.1 (45)	Selective (MA) for FPR1	Active for FPR1 and FPR2

*NA* no activity (on the basis of the limits of EC_50_ < 50 μM and efficacy ≥ 20%), *LA* low active (i.e. EC_50_ > 20 μM), *MA* moderate active (i.e. 10 μM ≤ EC_50_ ≤ 20 μM)

^a^ Ligand numbers match the original numbers^2, 6^; for experimental details see in refs. ^2 and ^^6^

^b^ Efficacy (in parentheses) is expressed as % of the response induced by 5 nM fMLF (FPR1) or 5 nM WKYMVm (FPR2) and is calculated only for ligands with EC_50_ < 30 μM

^c^ Previously reported potency of the ligands evaluated in HL-60 cells^2, 6^

^d^ See ref. ^10^ for experimental details, i.e. potency of the ligands in RBL-2H3 cells, measured through the collaboration with M.P. Giovannoni (University of Florence) and M.T. Quinn (Montana State University)

^e^ Potency of compound 17b for FPR2 in HL-60 cells. Due to the low efficacy (i.e. ≤ 20%, chosen cut-off for affinity)^2, 3, 6^, the ligand (as racemic mixture) has been considered a selective agonist for FPR1^2^

Taken together, these results indicate that the potency of ligands (as an indirect means to establish biological profiles, e.g. selectivity) for FPRs is strictly dependent on the cell type, where the receptors are expressed. This point was flagged by Reviewer 2 of ref. ^1^, who requested that the key experiments should be repeated in HEK-293 cells. Even though the transfected cells are useful tools to define a trend of binding for FPRs (and GPCRs in general), allowing the rapid preliminary screening of large compound libraries, no conclusive results on the apparent affinity/selectivity of such a ligand can be drawn from an investigation on a single cell line. To the best of my knowledge, previous ligand potency data in refs. ^2,3^ can only be reproduced if the same cell line and experimental settings are adopted. Thus, the result from Qin et al. is not “in contrast” with previous reports, but in line with typical issues encountered in GPCR testing, highlighting the requirement of new methods for the direct quantification of binding (e.g. by using optical ligands in single-molecule and/or super-resolution microscopy approaches). Additionally, these studies would also be beneficial to reduce discrepancies of activity data due to receptor density variability (i.e. differences in the number of GPCRs on the surface of transfected and primary cell lines), which is well known to limit the interpretation of biological profiles for ligands through their potency.

Most importantly, Qin et al. obtained compound 17b through a custom synthesis (from Anthem Bioscience) by adopting the synthetic route reported in ref. ^2^, which leads to a racemic mixture (i.e. 17b = (±)-5a)^2,3^. Consequently, compound 17b is a racemate and its biological effect is determined by two different chemical entities (i.e. stereoisomers) interacting with FPRs. Previous findings show that the two enantiomers, S-(+)-5a and R-(−)-5a^3^, have a different behaviour on FPR1 and FPR2. In particular, S-(+)-5a possesses a low micromolar potency on FPR1 (i.e. 3.2 μM) and only a moderate activity on FPR2 (i.e. 16.1 μM). In contrast, R-(−)-5a has almost the same potency on both FPR1 and FPR2 (i.e. 8.5 and 10.2 μM)^3^. Furthermore, a selective agonism of S-(+)-5a on FPR1 has been found by evaluating β-arrestin recruitment in CHO cells, the cell line used in the study by Qin et al.^1^. These events have established links with biased signals in cardioprotection^12^. In contrast, R-(−)-5a did not show FPR1- or FPR2-specificity in the same biochemical pathway (for details, see Table 3 in ref. ^3^).

Unfortunately, by focusing attention on the properties of the racemic mixture of compound 17b, Qin et al. do not take into consideration the intrinsic chemical diversity arising from the two enantiomers that will cause a misleading interpretation of the observed biological results. In this regard, various studies indicate that chirality plays a crucial role in ligand-FPR molecular recognition and related biological effects (see ref. ^3^ and refs. ^3^, 21–25 cited therein), demonstrating that enantio-resolved ligands possess markedly different affinities and potencies on FPRs, in agreement with results observed with GPCRs^13^. Moreover, enantio-selective interactions have also been shown to critically modulate the activity of ligands on various GPCRs in relation to biased effects^14,15^. Thus, evaluation of the influence of chirality is of primary importance in order to limit the reporting of ambiguous results. Pure enantiomers must be used for biological studies, especially in the case of intricate biochemical signalling.

Clearly, from the study of Qin et al. we can see that two different compounds interact with at least two receptor targets in the cells (i.e. FPR1 and FPR2). The authors fail to show any evidence for specific enantio-dependent outcomes on the possible FPR-biased agonism observed, this being in stark contrast to the previous observations. Without knowing which chemical entities are the main determinants for such a biased effect and which receptor is more likely to interact with each enantiomer, it is not possible to hypothesise FPR-biased agonism for compound 17b when presented as a racemic mixture. Given the well-known variability related to FPR testing and the high level of molecular diversity in ligand recognition (see ref. ^1^, and refs. ^3, 6^, 26 cited therein), it becomes evident that such an evaluation and quantification of biased effects for the single enantiomers is essential, in order to examine which is the more active (eutomer) and the less active (distomer) stereoisomer of compound 17b. In turn, the confirmation of the bias on FPR may facilitate our understanding of cardio-protection effects, under in cellulo and in vivo conditions.

In conclusion, the present comment emphasises that by evaluating biological profiles (e.g. selectivity) of ligands through potency on FPRs (and GPCRs), using such an indirect readout, one should first consider which results strongly depend on the transfected cell line where the receptors are expressed. Moreover, a high variability of ligand activity data has been also reported for FPRs when comparing species (e.g. human FPRs *vs* murine Fprs, expressed in different cell lines)^16^. With regard to the reported biased effects of racemate 17b, the experimental evidence provided in ref. ^1^ is insufficient to establish if an “underappreciated” FPR bias is present or whether it can be attributed to the extensive variation in such FPR–ligand interactions.

## References

[1] Qin CX (2017). Small-molecule-biased formyl peptide receptor agonist compound 17b protects against myocardial ischaemia-reperfusion injury in mice. Nat. Commun..

[2] Cilibrizzi A (2009). 6-Methyl-2,4-disubstituted pyridazin-3(2H)-ones: a novel class of small-molecule agonists for formyl peptide receptors. J. Med. Chem..

[3] Cilibrizzi A (2012). Synthesis, enantioresolution, and activity profile of chiral 6-methyl-2,4-disubstituted pyridazin-3(2*H*)-ones as potent *N*-formyl peptide receptor agonists. Bioorg. Med. Chem..

[4] Schepetkin IA (2014). Development of small molecule non-peptide formyl peptide receptor (FPR) ligands and molecular modeling of their recognition. Curr. Med. Chem..

[5] Li Y, Ye D (2013). Molecular biology for formyl peptide receptors in human diseases. J. Mol. Med..

[6] Giovannoni MP (2013). Further studies on 2-arylacetamide pyridazin-3(2H)-ones: Design, synthesis and evaluation of 4,6-disubstituted analogs as formyl peptide receptors (FPRs) agonists. Eur. J. Med. Chem..

[7] Atwood BK, Lopez J, Wager-Miller J, Mackie K, Straiker A (2011). Expression of G protein-coupled receptors and related proteins in HEK293, AtT20, BV2, and N18 cell lines as revealed by microarray analysis. BMC. Genom..

[8] Kenakin T, Christopoulos A (2013). Signalling bias in new drug discovery: detection, quantification and therapeutic impact. Nat. Rev. Drug Discov..

[9] Eisenstein M (2009). GPCRs: insane in the membrane. Nat. Methods.

[10] Kirpotina LN (2010). Identification of novel small-molecule agonists for human formyl peptide receptors and pharmacophore models of their recognition. Mol. Pharmacol..

[11] Sogawa Y, Shimizugawa A, Ohyama T, Maeda H, Hirahara K (2009). The pyrazolone originally reported to be a formyl peptide receptor (FPR) 2/ALX–selective agonist is instead an FPR1 and FPR2/ALX dual agonist. J. Pharmacol. Sci..

[12] Carr R (2016). β-arrestin–biased signaling through the β2-adrenergic receptor promotes cardiomyocyte contraction. Proc. Natl Acad. Sci. USA.

[13] Seifert R, Dove S (2009). Functional selectivity of GPCR ligand stereoisomers: new pharmacological opportunities. Mol. Pharmacol..

[14] Manglik A (2016). Structure-based discovery of opioid analgesics with reduced side effects. Nature.

[15] Woo AY (2014). Tyrosine 308 is necessary for ligand-directed Gs protein-biased signaling of β2-adrenoceptor. J. Biol. Chem..

[16] Vergelli C (2016). 2-Arylacetamido-4-phenylamino-5-substituted pyridazinones as formyl peptide receptors agonists. Bioorg. Med. Chem..

